# Effect of oral beta-lactam dosing on outcomes of bacteremic Gram-negative urinary tract infections: a real-world analysis

**DOI:** 10.1128/spectrum.01704-25

**Published:** 2025-12-31

**Authors:** Stephanie S. May, John J. Veillette, Allison M. Butler, Jared Olson, Sameer Alzaidi, Brandon J. Webb

**Affiliations:** 1 Infectious Diseases Telehealth Service, Intermountain Health, Murray, Utah, USA; 2 Department of Pharmacy, Intermountain Medical Center 98078, Murray, Utah, USA; 3 Statistical Data Center, Intermountain Health, Murray, Utah, USA; 4 Department of Pharmacy, Primary Children’s Hospital, Salt Lake City, Utah, USA; 5 Division of Pediatrics, University of Utah, Salt Lake City, Utah, USA; 6 King Abdul Aziz Specialist Hospital, Taif, Makkah, Saudi Arabia; 7 Division of Clinical Epidemiology and Infectious Diseases, Intermountain Medical Center 98078, Murray, Utah, USA; JMI Laboratories, North Liberty, Iowa, USA

**Keywords:** Gram-negative bacteremia, oral, beta-lactam

## Abstract

Among 452 adult patients with Gram-negative bacteremia from a urinary tract infection source who received oral step-down therapy with highly bioavailable oral beta-lactam, we observed no difference in 60-day recurrence in patients treated with higher consensus-recommended dosing compared to lower, standard dosing (odds ratio = 0.76 [95% confidence interval = 0.43–1.32]), although the directionality of the effect was favorable. The study was limited by inadequate sample size and confounding. Randomized or large observational comparative effectiveness studies are needed.

**IMPORTANCE:**

Definitive treatment of Gram-negative bacteremia with oral antibiotic agents is not one size fits all. Use of oral fluoroquinolones and sulfamethoxazole/trimethoprim is evidence-based but carries risk for adverse events. Data supporting the use of oral beta-lactams are mixed, likely in part due to variable agent selection and dosing. This analysis shows that higher, consensus-recommended dosing of highly bioavailable beta-lactam antibiotics was not significantly different from patients treated with lower dosing on 60-day recurrence. Our study was limited by the number of patients with discrete MICs and overinfluence of complicated urinary tract infection on outcomes.

## OBSERVATION

Oral fluoroquinolones (FQs) and trimethoprim-sulfamethoxazole (TMP-SMX) are preferred step-down options for Gram-negative bacteremia from a urinary tract infection source (GNBSI-UTI) (1–3). However, both are limited by toxicity concerns and increasing *Enterobacterales* resistance rates. High-bioavailability oral beta-lactams (HBBLs) are well tolerated and exhibit activity against *Enterobacterales* but have been associated with inferior efficacy to FQs/TMP-SMX in cystitis trials (4–6) and some retrospective GNBSI studies (7–10). Importantly, the latter studies were limited by low HBBL dosing, and it is unknown whether higher consensus-recommended dosing (HCD) of HBBLs (1) could mitigate treatment failures. Consensus-recommended dosing of oral beta-lactams is higher for treatment of bloodstream infections than initial FDA-approved dosing, which was based on common non-bacteremic infections. Higher dosing has been recommended by consensus guidelines to optimize total antibiotic exposure.

Data are needed to assess the impact of HBBL dosing on GNBSI-UTI outcomes. Therefore, we conducted a multicenter observational cohort study of adult inpatients with GNBSI-UTI due to *Escherichia coli*, *Klebsiella pneumoniae*, or *Klebsiella oxytoca* who received a single susceptible HBBL (amoxicillin, amoxicillin-clavulanate, or cephalexin) as definitive therapy between January 2016 and December 2022. Methods for electronic and manual data extraction from the Intermountain Health enterprise data warehouse were previously described (7, 8). Patients had to receive 1–7 days of an effective IV beta-lactam (IVBL) before step down to HBBL. Patients with polymicrobial or concomitant infections, prolonged time to effective therapy (>24 h), pregnancy, in-hospital mortality, discharge with hospice, transfer to an outside facility, or loss to follow-up after discharge (no further notes or follow-up visits in the electronic medical record [EMR]) were excluded.

For the primary analysis, patients were assessed in two groups: HCD (amoxicillin 1,000 mg PO q8h, amoxicillin/clavulanic acid 875–1,000 mg PO q8h, or cephalexin 1,000 mg PO q6h, with adjustments for renal impairment) or lower dosing (LD), which was any dosing less than HCD. To test the mechanistic hypothesis that any differences in outcome in the HCD group may have been attributable to optimal target attainment relative to MIC given the higher-than-conventional dosing, we conducted a sensitivity analysis to investigate whether HBBL exposure (time over MIC in a 24-h dosing interval [(%T > MIC]) was associated with recurrence. For the sensitivity analysis, we used published pharmacokinetic equations to predict individual elimination rate constants and volumes of distribution, which were then used to estimate percent time over MIC (%T > MIC) for each patient (11–13). Protein binding was assumed to be 10% for cephalexin and 20% for amoxicillin (14, 15). Cefazolin MIC was used as a surrogate for cephalexin, and ampicillin MIC was used for amoxicillin per CLSI guidance (16). A discrete MIC value from the blood culture isolate was used when available (i.e., when susceptibility testing was performed with BD Phoenix panels). Otherwise, a worst-case scenario MIC was assumed for calculations based on the reported estimated value (i.e., MIC ≤ 2 was assigned an MIC value of 2).

The primary outcome was 60-day recurrence defined as positive blood or urine culture for the same organism, excluding patients with only a positive urine culture who lacked urinary symptoms (i.e., asymptomatic bacteriuria). HCD and LD groups were compared using Fisher’s exact test (categorical data) or the Mann-Whitney *U*-test (continuous data). A multivariate logistic regression was used to determine the relationship between HBBL dosing and recurrence controlling for gender, Pitt Bacteremia score, days of effective IV antibiotics, and complicated UTI [cUTI] source (defined as structural or functional urologic abnormality). For the sensitivity analysis, we used a multivariate Cox proportional hazards model to determine the relationship between %T > MIC and time to 60-day recurrence. We also conducted a subgroup analysis limited to patients with discrete MICs.

Out of 2571 patients screened with GNBSI-UTI, 452 met inclusion criteria, and 166 out of 452 (36.7%) received HCD. Most patients were female (342 [76%]), and >65 years of age (270 [59%])]. There were several notable imbalances between groups including higher median age, comorbidities, cUTI source, severity of illness, and days of IVBL therapy (median 4 vs 3 days, *P* = 0.015) in the HCD group (Table 1). Unadjusted rates of 60-day recurrence favored the HCD group (both overall and for a subgroup limited to patients with cUTI only), but differences were not statistically significantly different (Table 1).

**TABLE 1:** High-bioavailability beta-lactam (HBBL) higher consensus-recommended dosing (HCD) versus lower dosing (LD) groups for Gram-negative bacteremia from a urinary tract infection source (GNBSI-UTI)^*g*^

	All patients (*N* = 452)	HBBL HCD (*n* = 166)	HBBL LD (*n* = 286)	*P* value^*h*^
Age in years, median (IQR)	71 (55–80)	73 (57–81)	70 (54–78)	**0.032**
Female^*a*^	342 (76)	126 (76)	216 (76)	1.00
Charlson comorbidity score, median (IQR)	6 (3–9)	6 (3–9)	5 (3–8)	0.078
Complicated UTI source^*b*^	214 (47)	93 (56)	121 (42)	**0.006**
History of kidney stones	62 (14)	30 (18)	32 (11)	**0.047**
Urinary retention or neurogenic bladder	39 (9)	17 (10)	22 (8)	0.387
Benign prostatic hypertrophy	35 (8)	11 (7)	24 (8)	0.586
Chronic urinary incontinence	33 (7)	18 (11)	15 (5)	**0.038**
Cancer or mass of bladder/prostate/kidney	27 (6)	11 (7)	16 (6)	0.683
Baseline urinary catheterization	27 (6)	11 (7)	16 (6)	0.683
Urologic stricture, stenosis, or obstruction	20 (4)	8 (5)	12 (4)	0.814
Baseline stent or nephrostomy tube	9 (2)	2 (1)	7 (2)	0.496
Urologic procedure in previous 2 weeks	8 (2)	3 (2)	5 (2)	1.00
Cystocele or urologic fistula	7 (2)	5 (3)	2 (1)	0.106
Other urologic abnormalities^*c*^	4 (1)	2 (1)	2 (1)	0.627
Index bacteremia characteristics				
Active kidney/ureteral stone	69 (15)	31 (19)	38 (13)	0.137
Hydronephrosis or hydroureter	63 (14)	31 (19)	32 (11)	**0.034**
Ureteral stent or nephrostomy tube placed	38 (8)	21 (13)	17 (6)	**0.021**
Admitted to hospital^*d*^	369 (82)	146 (88)	223 (78)	**0.008**
Length of stay in hours, median (IQR)	67 (7–92)	68 (45–97)	65 (5–89)	**0.044**
Pitt bacteremia score, median (IQR)	1 (1–3)	2 (1–3)	1 (1–2)	0.099
Admitted to intensive care unit	89 (20)	41 (25)	48 (17)	**0.049**
Received vasopressors	43 (10)	25 (15)	18 (6)	**0.004**
Achieved clinical stability within 3 days^*e*^	432 (96)	159 (96)	273 (95)	1.00
*Escherichia coli* as causative organism^*f*^	401 (89%)	151 (91)	250 (87)	0.283
Antibiotic treatment				
Days of active IVBL therapy, median (IQR)	3 (3–4)	4 (3–5)	3 (2–4)	**0.015**
Days of active oral HBBL therapy, median (IQR)	9 (7–10)	9 (7–11)	9 (7–10)	1.00
Cephalexin				
1,000 mg q6h	56 (12)	56 (34)		
1,000 mg q8h or 500 mg q6h (CrCl ≤ 50)	47 (10)	47 (28)		
lower dosing	190 (42)		190 (66)	
Amoxicillin				
1,000 mg q8h	28 (6)	28 (17)		
1,000 mg q12h or 500 mg q8h (CrCl ≤ 30)	12 (3)	12 (7)		
lower dosing	16 (4)		16 (6)	
Amoxicillin-clavulanate				
875–125 mg q8h	5 (1)	5 (3)		
500–125 mg or 875–125 mg q12h (CrCl ≤ 30)	18 (4)	18 (11)		
Lower dosing	80 (18)		80 (28)	
60-day recurrence				
All patients (*n* = 452)	65/452 (14)	22/166 (13)	43/286 (15)	0.677
Complicated UTI source only (*n* = 214)	46/214 (21)	16/93 (17)	30/121 (25)	0.240

^*a*^ Values are represented as number (percent) unless otherwise specified (e.g., median, IQR).

^*b*^ Structural or functional urologic abnormality (patients could have more than one); remaining patients had no evidence of urologic abnormality on manual chart review.

^*c*^ Other urologic abnormalities included: bladder sling (*n* = 1), chronic interstitial cystitis (*n* = 1), and urostomy (*n* = 2).

^*d*^ Remaining patients were discharged from the emergency department after a single IVBL dose.

^*e*^ Clinical stability was defined as afebrile and hemodynamically stable.

^*f*^ Remaining patients had bacteremia due to *Klebsiella pneumoniae* (*n* = 49) or *Klebsiella oxytoca* (*n* = 2).

^*g*^ CrCl, creatinine clearance (mL/min); HBBL, high-bioavailability oral beta-lactam; HCD, higher consensus-recommended dosing; IQR, interquartile range; IVBL, intravenous beta-lactam; LD, lower dosing; UTI, urinary tract infection.

^*h*^ Bolded values are statistically significant, *P* < 0.05.

HCD was not significantly associated with 60-day recurrence in the multivariate model (odds ratio [OR] = 0.76 [95% confidence interval [CI] = 0.43–1.32]), and cUTI was noted to have a strong association with recurrence in the model (OR = 2.36 [1.33–4.28]). Similar results were observed in the sensitivity analysis: %T > MIC was not significantly associated with time to 60-day recurrence (hazard ratio [HR] = 1.00 [95% CI = 0.98–1.01]) with heavy influence of cUTI on the recurrence endpoint (HR = 3.80 [95% CI = 1.20–11.98]). When cUTI was coded as an interaction term with %T > MIC in the model, results were similar.

When we limited the sensitivity analysis to 97 out of 452 patients (21.7%) with discrete MICs (i.e., omitted 355 patients where a worst-case scenario MIC was assumed), %T > MIC was significantly associated with time to 60-day recurrence in univariate analysis (HR = 0.97 [95% CI = 0.95–1.00]), but sample size was insufficient to perform a multivariate analysis. Given the observed effect size, we estimated that at least 200 patients with discrete MICs would be required to detect a possible difference in the multivariate model with 90% power.

Our data suggest a possible signal toward lower 60-day recurrence when HBBLs are dosed according to HCD in step-down therapy for GNBSI-UTI; however, our sample size was inadequately powered and overly influenced by the confounding of cUTI to make conclusive inferences. It is worth noting that in our previous GNBSI-UTI study, even when HBBL were dosed according to HCD, the crude recurrence rate (17.2%) was higher than rates observed for FQs (7.3%) or TMP-SMX (9.3%) (7, 8). Randomized controlled or pragmatic comparative effectiveness trials, or much larger observational cohorts using causal methodologies would be necessary in order to establish whether HCD HBBL therapy may be equivalent to FQs or TMP-SMX as step-down agents for susceptible isolates in patients with favorable toxicity risk profiles.

This study had several limitations. First, unmeasured confounders such as patient compliance or undocumented post-discharge change in antibiotics might have impacted the results. Second, we were unable to capture the timing of post-discharge urologic procedures for source control (e.g., lithotripsy with ureteral stent removal) because these often took place outside the IH system. Third, readmissions and recurrences outside IH were not captured, and we were unable to distinguish between recurrence versus new infection during retrospective review. Finally, only 36.7% of patients in our study received HCD, and only 21.7% had a discrete MIC reported in the EMR (for surrogate IVBLs to infer susceptibility of oral agents), which limited our ability to associate predictors for HCD or to detect significant relationships between HBBL exposure and outcomes. Additionally, the antimicrobial susceptibility testing procedures varied widely across different facilities and across the 6-year study period. Real-world MIC assignment also comes with inherent limitations, including the MIC precision of automated systems.

## SUPPLEMENTAL MATERIAL

Supplemental material spectrum.01704-25-s0001.docx AST methods and MIC distribution.

ASM does not own the copyrights to Supplemental Material that may be linked to, or accessed through, an article. The authors have granted ASM a non-exclusive, world-wide license to publish the Supplemental Material files. Please contact the corresponding author directly for reuse.
