# Supplementary material for: Effect of oral beta-lactam dosing on outcomes of bacteremic Gram-negative urinary tract infections: a real-world analysis
Source: Microbiol Spectr. 2025 Dec 31;14(2):e01704-25. doi: 10.1128/spectrum.01704-25 (PMC12889076; doi:10.1128/spectrum.01704-25)
Supplement: Supplemental material — AST methods and MIC distribution. [file spectrum.01704-25-s0001.docx]

Antimicrobial Susceptibility Testing Methods

Over the six-year study period, antimicrobial susceptibility testing methods varied. Most patients included in this multi-facility cohort had antimicrobial susceptibility testing performed at a central laboratory. In 2016, the central laboratory implemented BD Phoenix (BD Diagnostic Systems) for blood culture antimicrobial susceptibility testing using Gram-negative panels. Urine culture susceptibility testing was performed on MicroScan Walkaway (Beckman Coulter). The urine culture panels were updated in 2016 (Neg Urine Combo 61) and again in 2019 (Neg Urine Combo 85). Specific data regarding antimicrobial susceptibility testing method for each patient were unfortunately not stored in the electronic health record, so a limitation is the inability to provide more context than historical central laboratory procedures. However, these methods applied to most isolates during the study period. Precise minimum inhibitory concentration (MIC) data were lacking for many isolates as MICs were often below the limit of detection, so a sensitivity analysis was performed to include only isolates with discrete MICs. infer susceptibility for oral antibiotics (eg, cephalexin and amoxicillin) based on surrogate intravenous antibiotics reported on the panel (eg, cefazolin and ampicillin, respectively). Because of this limitation, we preplanned an analysis limited to isolates confirmed to be susceptible by current Clinical and Laboratory Standards Institute (CLSI) breakpoints

Discrete MIC Distribution


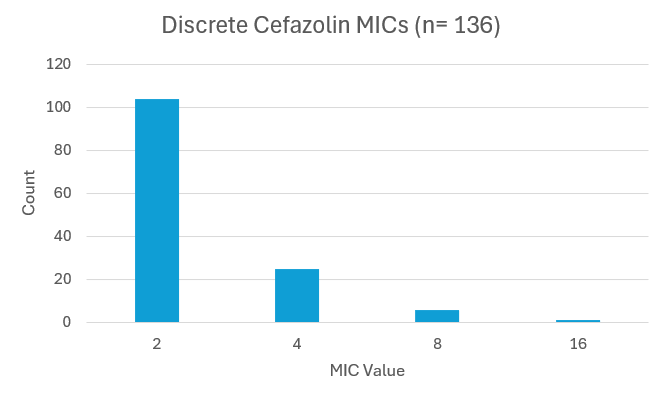

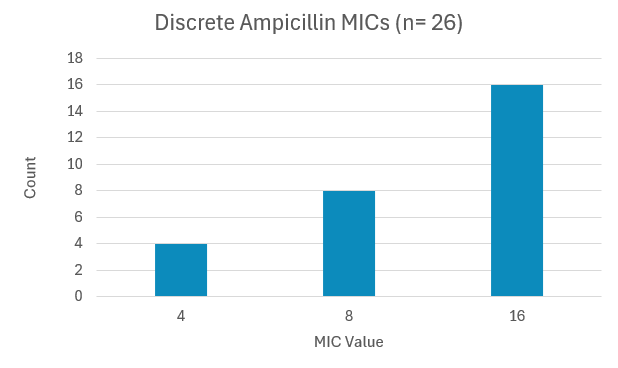


Summary of inexact MIC data: There were 131 isolates with cefazolin MIC reported as ≤1, 82 isolates with cefazolin MIC reported as ≤2, 3 isolates with cefazolin MIC reported as ≤4, 61 isolates with cefazolin MIC reported as ≤8, 1 isolate with cefazolin MIC reported as >16, and 1 isolate with cefazolin MIC reported as > 32. There were 84 isolates with ampicillin MIC reported as ≤2, 93 isolates with ampicillin MIC reported as ≤4, 71 isolates with ampicillin MIC reported as ≤8, 172 isolates with ampicillin MIC reported as >16, and 2 isolates with ampicillin MIC reported as > 32.
